# Small molecule tyrosine kinase inhibitors modulated blood immune cell counts in patients with oncogene-driven NSCLC

**DOI:** 10.1186/s40364-021-00324-6

**Published:** 2021-09-06

**Authors:** Weijie Ma, Jie Zeng, Shuai Chen, Yue Lyu, Kyra A. Toomey, Chinh T. Phan, Ken Y. Yoneda, Tianhong Li

**Affiliations:** 1 Division of Hematology/Oncology, Department of Internal Medicine, University of California Davis School of Medicine, University of California Davis Comprehensive Cancer Center, 4501 X Street, Suite 3016, Sacramento, California 95817 USA; 2 Department of Respiratory Medicine, Shanghai Tenth People’s Hospital, Tongji University School of Medicine, Shanghai, 200072 People’s Republic of China; 3 Division of Biostatistics, Department of Public Health Sciences, University of California, Davis, California USA; 4 College of Agricultural and Environmental Sciences, University of California Davis, Davis, California 95616 USA; 5 Division of Pulmonary, Critical Care, and Sleep Medicine, Department of Internal Medicine, University of California Davis, Sacramento, California USA; 6 Medical Service, Pulmonology, Veterans Affairs Northern California Health Care System, Mather, California USA; 7 Medical Service, Hematology and Oncology, Veterans Affairs Northern California Health Care System, Mather, California USA

**Keywords:** Tyrosine kinase inhibitor, Peripheral blood mononuclear cells, Immune cells, Oncogenic-driven, NSCLC, In vitro cytotoxicity, Malignant pleural effusion

## Abstract

**Background:**

Lack of biomarkers and in vitro models has contributed to inadequate understanding of the mechanisms underlying the inferior clinical response to immune checkpoint inhibitors (ICIs) in patients with oncogene-driven non-small cell lung cancer (NSCLC).

**Methods:**

The effect of small molecule tyrosine kinase inhibitors (TKIs) on peripheral blood mononuclear cells (PBMCs) in 34 patients with oncogene-driven NSCLC (cohort A) was compared with those from 35 NSCLC patients without oncogene-driven mutations received ICI (cohort B) or from 22 treatment-naïve NSCLC patients (cohort C). Data for each blood biomarker were summarized by mean and standard deviation and compared by Wilcoxon rank sum tests or Kruskal-Wallis tests with significance at 2-sided *p* value < 0.05. Co-culture of PBMCs and pleural effusion-derived tumor cells from individual patients with oncogene-driven NSCLC was used to determine the in vitro cytotoxicity of TKI and ICI.

**Results:**

Except for low CD3% in cohort A, there were no significant differences in other 12 blood biomarkers among the 3 cohorts at baseline. TKI treatment in cohort A was associated with significant increase in CD3% and decrease in total and absolute neutrophils (*p* < 0.05). In cohort B, patients with good clinical response to ICI treatment (*N* = 18) had significant increases in absolute lymphocyte counts (ALCs), CD4 and/or CD8 cell counts. Conversely, those patients with poor clinical response to ICI (*N* = 17) had significant decreases in these cell counts. Of the 27 patients with pre- and post-treatment blood samples in cohort A, 11 had poor clinical response to TKIs and decreased lymphocyte counts. Of the remaining 16 patients who had good clinical response to TKI therapy, 10 (62.5%) patients had decreased, and 6 (37.5%) patients had increased lymphocyte counts. Multicolor immunophenotyping of PBMCs revealed ICI treatment activated additional immune cell types that need further validation. We confirmed that TKI treatment could either antagonize or enhance the effect of ICIs in the co-culture assay using patient’s tumor cells and PBMCs.

**Conclusions:**

To the best of our knowledge, this is the first study showing that TKIs can have various effects on blood immune cells, which may affect their response to ICIs. Further validation of the blood biomarker and in vitro assay is warranted.

## Background

Immune checkpoint inhibitors (ICIs) have revolutionized the diagnosis and treatment for patients with locally advanced or metastatic non-small cell lung cancer (mNSCLC). However, ICIs have low or inferior clinical efficacy compared to chemotherapy in patients with epidermal growth factor receptor *(EGFR)*-mutant or anaplastic lymphoma kinase *(ALK)*-rearranged mNSCLC [1–3]. This low clinical efficacy of ICIs in *EGFR*-mutant or *ALK*-rearranged mNSCLC remains even when their tumors had high Programmed death-ligand 1 (PD-L1) immunohistochemistry (IHC) expression [4]. Furthermore, ICI treatment has been associated with increased incidence and severity of interstitial lung disease and immune-mediated adverse effects (including pneumonitis, colitis and hepatitis) when they are in sequential or concurrent use with small molecule tyrosine kinase inhibitors (TKIs) in patients with *EGFR*-mutant or *ALK*-rearranged mNSCLC [5]. Thus, mNSCLC patients with oncogene-driven mutations have been excluded in the first-line ICI trials except in the case of atezolizumab in IMpower 150 study [6]. The addition of atezolizumab to carboplatin, paclitaxel and bevacizumab (CPB) had superior clinical activity compared to CPB in a small cohort of patients with *EGFR*-mutant or *ALK*-rearranged cohorts after first-line TKI therapy. The key effect of angiogenesis inhibitor bevacizumab is postulated, which has synergism with each of the other 3 components. Recently, the updated report of PACIFIC [7] and retrospective analysis on durvalumab consolidation for patients with stage III NSCLC [8, 9] suggested that durvalumab might have limited clinical efficacy in the small subset of patients with *EGFR*- or *HER2*-mutant NSCLC.

ICIs are designed to activate exhausted tumor-reactive T cells, which are responsible for killing tumor cells. Current data suggest that the presence of high membranous PD-L1 IHC staining on tumor cells and the presence of intratumoral PD-1 expressing tumor infiltrating lymphocytes (TILs) in the tumor microenvironment (TME) are favourable prognostic factors and the best predictive factors of clinical response to ICIs [10]. The presence of a T-cell inflamed gene expression profile (GEP) in addition to PD-L1 IHC has improved the prediction of favourable clinical response to ICIs [10]. ICIs could increase the number of absolute lymphocyte counts (ALCs), restore the function in exhausted CD8+ T cells and induce phenotypically and functional changes of effector immune cells [11]. Several mechanisms have been postulated for the underlying mechanisms by which patients with oncogene-driven NSCLC do not derive clinical benefit from ICI [12]. These include low PD-L1 expression on tumor cells and TILs, low tumor mutation burden (TMB) and immune escape using other immune checkpoints in patients with *EGFR*-mutant NSCLC [13–16]. However, these mechanisms do not explain the inferior clinical response observed in patients with oncogene-driven NSCLC and high PD-L1 expression [17]. Further, functional studies have been hampered by insufficient paired tumor specimens before and after treatment. Along with, a lack of relevant human NSCLC models that can simulate the interaction and delineate the mechanisms of ICI with TKI, chemotherapy, and/or bevacizumab.

Increasingly, liquid biopsy with blood and malignant body fluids have been used to provide a minimally invasive way to study tumor biology and monitor dynamic changes of molecular and immune biomarkers during cancer treatment [18]. Previous studies have shown that pre-treatment low ALCs (< 600–1200 cells/μL) were associated with decreased progression-free survival (PFS) and overall survival (OS) to ICI in NSCLC patients [19–22]. Dynamic changes of ALCs after ICI treatment were also associated with clinical response. Post-ICI treatment low ALCs (< 700–900 cells/μL) were associated with decreased PFS and OS in patients with advanced solid cancer types including NSCLC [20, 23]. Immunophenotypic analysis of circulating immune cells revealed increases in circulating proliferating CD4+ and CD8+ T cells at 2 weeks after durvalumab treatment [24]. Assessing the function of peripheral T-cell subclones, particularly the T-cell receptor (TCR) clonality and activity to clonal neoantigens, have also been explored as a predictive biomarker for response to ICI [24, 25]. Furthermore, high derived neutrophil-to-lymphocyte ratio (dNLR) was associated with poor prognosis in patients with advanced NSCLC treated with durvalumab [26]. However, the effect of small molecule TKIs on these blood immune cells in patients with oncogene-driven NSCLC are unknown. The objective of this study was to determine the effect of small molecule TKIs on blood immune cells in patients with oncogene-driven NSCLC. We also explored the feasibility of using tumor cells from malignant pleural effusion and patient’s own peripheral blood mononuclear cells (PBMCs) for in vitro evaluation of the effect of targeted therapy and ICIs.

## Methods

### Study patients and biospecimen collection

Lung cancer patients receiving care at an academic institution between March 2017 and March 2021 were retrospectively identified through chart review if their tumors had been tested for tumor genomic profiling by a clinical next generation sequencing (NGS) assay under an Institutional Review Board (IRB) approval protocol (University of California, Davis Protocol No. 937274). Patients whose tumors had at least one driver oncogene, defined as *EGFR, MET* exon 14 skip or *ERBBR2 (HER2)* mutation, *ALK, ROS1* or *RET* fusions, were defined as oncogene-driven NSCLC according to National Comprehensive Cancer Network (NCCN) guidelines. Demographic information, clinical genomic sequencing results, and complete blood cell counts with differentials were abstracted from electronic medical records. Fresh biofluids (blood and malignant pleural effusion if available) were collected via an IRB approved protocol (University of California, Davis Protocol No. 226210). Multiple samples from the same patients at different time points were collected during the disease course. Cell pellets from malignant pleural effusion were washed three times in 1x phosphate-buffered saline (PBS) buffer containing 0.2% BSA and 10 mM ethylenediamine tetraacetic acid (EDTA) and resuspended in 10 ml of the 1x PBS buffer for use. The samples were handled under strict operating procedures for collection, processing, and storage to minimize the variation in handling of samples.

### Immunophenotyping of PBMCs by flow cytometry

Fresh or thawed PBMCs were immunophenotyped for T cell subsets and reported as percentages of total PBMCs and as percentages of total T cells in the case of T cell subtypes. Specifically, PBMCs were stained with well-characterized antibodies against markers of interest, including CD3, CD4 plus CD8 according to standard protocols. Results were analyzed using a BD Fortessa multi-color flow cytometer and FlowJo 7.6.1 program (Ashland, OR). Further, immunophenotypic changes of major innate and adaptive immune cells of ICI were evaluated using a minimum of a million PMBCs collected before and after cancer therapy for individual lung cancer patients by a 24-color antibody panel using the “Aurora” Spectral cytometer (Cytek Biosciences, CA). The data analysis and statistical evaluations of this complex data set were performed using the Cytobank (Cytobank Inc., CA) [27].

### Data collection and statistical analysis

Data were summarized according to frequency and percentage for qualitative variables, and by mean ± standard deviation (SD) for quantitative variables unless noted otherwise. The 95% confidence interval for survivals was calculated using the exact binomial distribution. For each of the 13 blood cell types, the cell counts were summarized using mean and SD for pre- and post-treatment in cohort and response subgroups. Wilcoxon rank sum tests were used to compare two groups (or Kruskal-Wallis tests for three cohorts). Two-sided *P* < 0.05 was used to determine statistical significance. Due to the exploratory nature, adjustment for multiplicity was not performed for the types of blood cells [28]. Statistical analyses were carried out using SAS version 9.4 (SAS Institute, Cary, NC).

According to the National Cancer Institute (NCI) Common Terminology Criteria for Adverse Events (CTCAE) Version 5.0 (http://ctep.info.nih.gov), lymphopenia (< 1000 cells/μl) was defined as grade 1: ALCs 800–999 cells/μL; grade 2: ALCs 500–799 cells/μL; grade 3: ALCs 200–499 cells/μL and grade 4: ALCs < 200 cells/μL. Best response to systemic therapies, defined as a complete or partial response (CR or PR), stable disease (SD) or progression disease (PD) achieved to cancer treatment, was assessed using Response Evaluation Criteria in Solid Tumors (RECIST) version 1.1 [29]. PFS was measured as the time from the first administration of a cancer therapy to progression defined by RECIST1.1, or death due to any cause. Patients alive without progression at the time of analysis were censored at the initiation of a new therapy or last follow-up. Good clinical response was defined in patients who achieved a CR, PR, PFS exceeding the reported median PFS for each targeted therapy. OS was measured as the time from the first administration of a cancer therapy to death due to any cause. Patients alive at the time of analysis were censored at the initiation of a new therapy or last follow-up. Survival data were estimated using the Kaplan–Meier method and compared using the log-rank test in each cohort and response subgroups. For the blood cell types significant in univariable survival analysis, multivariable survival analysis was further conducted using Cox proportional hazards models, by further adjusting for age, gender, race and histology in the model of each cell type.

### Co-culture of patient’s tumor cells and PBMCs and growth inhibition by the MTS assay

H1975 and primary tumor cells isolated from the malignant pleural effusion of NSCLC patients with oncogene-driven mutations were seeded in 96-well plates at a density of 5 × 10^3^ cells/well overnight. Human PBMCs from 20 ml of blood from the same patients were first purified by Ficoll-Paque and washed twice in 1x PBS. The acquired PBMCs were counted and added in a 2:1 ratio to the seeded tumor cells on the 96-well plates for co-culturing. After 8–12-h incubation, the cells were treated with various concentrations (0, 0.001, 0.01, 0.1, 1, 10 μM) of an TKI (osimertinib or alectinib) and/or an ICI (nivolumab (10 μg/mL), or atezolizumab (10 μg/mL) as indicated. The MTS assay was performed and analyzed as described previously [30, 31]. Specifically, after 72-h incubation, the MTS solution (3-(4,5-dimethylthiazol-2-yl)-5-(3-carboxymethoxyphenyl)-2-(4-sulfophenyl)-2H-tetrazolium) was added and the cell viability was measured by the absorbance at 490 nm using a microplate reader (SpectraMax M3, Molecular Devices, USA). Untreated cells served as a control. Results were shown as the average cell viability ± SD [(ODtreat−ODblank)/(ODcontrol−ODblank) × 100%] of triplicate wells. Data were presented as the mean ± SD unless noted otherwise. All the experiments were performed in triplicate. Statistical analysis was performed using Graph Prism software (Version 8.21). Two-sided, *P* < 0.05 was considered statistically significant.

## Results

### Patients’ characteristics and baseline blood cell counts

**Fig. 1:**
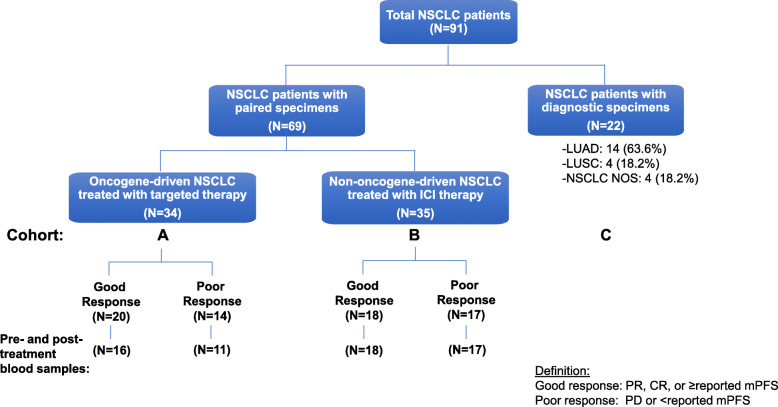
Flow chart for study patients. Abbreviations: NSCLC, non-small cell lung cancer; LUAD, lung adenocarcinoma; LUSC, lung squamous cell carcinoma; NOS, not other specified; CR, complete response; mPFS, median progression free survival; PD, progression disease; PR, partial response

**Fig. 2:**
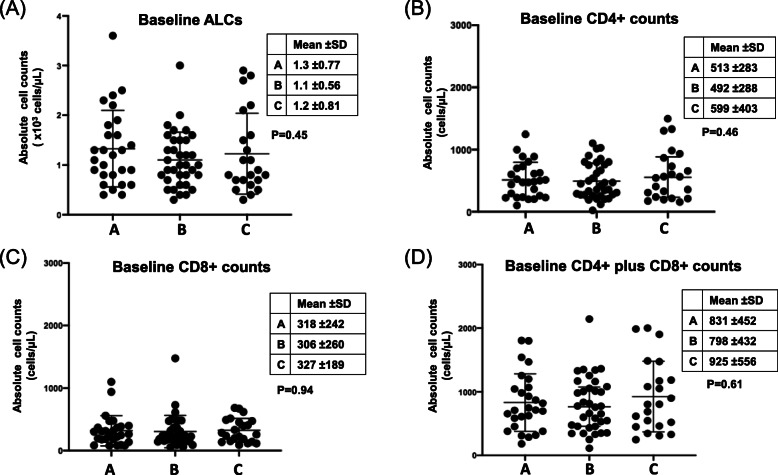
Assessment of baseline blood counts in patients with NSCLC. Baseline ALCs (**A**), CD4 (**B**), CD8 (**C**) and CD4 plus CD8 (**D**) cell counts for different cohorts are illustrated. Bar represents mean and SD. P-values were tested by the Kruskal-Wallis test

A total of 91 NSCLC patients were included in this study as illustrated in the flow chart (Fig. 1), 34 had blood samples after TKI treatment and 27 patients also had pre-treatment blood samples (cohort A), 35 had blood samples before and after ICI treatment (cohort B), and 22 had blood samples collected at diagnosis only (cohort C). Table 1 summarizes the clinicopathological and molecular characteristics of all study patients. Consistent with known clinicopathological features of oncogene-driven NSCLC, cohort A had more women (65%), Asians (50%), and lung adenocarcinomas (97%) compared to cohorts B and C. Driver oncogene mutations included *EGFR* mutations (*N* = 24, 70.6%), *ALK* fusions (*N* = 3, 8.8%), *HER2* alterations (*N* = 3, 8.8%), *MET* alterations (*N* = 3, 8.8%) and *RET* fusion (*N* = 1, 2.9%). Details of 34 patients with oncogene-driven NSCLC are summarized in Table 2. Of 13 blood cell types, there were no significant differences in the baseline cell counts among the 3 cohorts of NSCLC patients except CD3% was significantly lower in cohort A (Table 3). However, we did not observe any significant differences among the absolute cell counts of major lymphocyte subtypes. Figure 2 illustrates the cell counts of 4 major blood immune cell types: ALCs (1.3 ± 0.77, 1.1 ± 0.56, and 1.2 ± 0.81 × 10^3^ cells/μL for cohort A, B, C, respectively, *P* = 0.45) (Fig. 2a), CD4 counts (513 ± 283, 492 ± 288, and 599 ± 403 cells/μL for cohort A, B, C, respectively, *P* = 0.46) (Fig. 2b), CD8 (318 ± 242, 306 ± 260, and 327 ± 189 cells/μL for cohort A, B, C, respectively, *P* = 0.94) (Fig. 2c), and CD4 plus CD8 counts (831 ± 452, 798 ± 432, and 925 ± 556 cells/μL for cohort A, B, C, respectively, *P* = 0.61) (Fig. 2d). We did not find any correlation between immune cell counts and PD-L1 IHC expression or TMB (non-synonymous, somatic mutations per megabase, Mut/Mb) expression on tissues (Table 2).

**Table 1:** Clinicopathological and molecular characteristics of study patients

Category	Group A	Group B	Group C
**No. Patient**	34	35	22
**Age: median (range)**	67.3 (43–85)	69.9 (45–92)	67.1 (55–81)
**Gender: female N (%)**	22 (64.7%)	17 (48.6%)	10 (45.5%)
**Race/ethnicity: N (%)**			
**Non-Hispanic White**	16 (47.1%)	32 (91.4%)	17 (77.3%)
**Hispanic**	1 (2.9%)	1 (2.9%)	4 (18.2%)
**Asian**	17 (50.0%)	2 (5.7%)	1 (4.5%)
**Histology: N (%)**			
**LUAD**	33 (97.1%)	25 (74.4%)	14 (63.6%)
**LUSC**	1 (2.9%)	10 (28.5%)	4 (18.2%)
**NSCLC-NOS**	0 (0%)	0 (0%)	4 (18.2%)
**Driver oncogene mutations:**	34 (100%)	0 (0%)	7 (31.8%)
***EGFR*** **mutations**	24 (70.6%)	0 (0%)	3 (13.6%)
***ALK*** **fusions**	3 (8.8%)	0 (0%)	2 (9.1%)
***HER2*** **alterations**	3 (8.8%)	0 (0%)	1 (4.5%)
***MET*** **alterations**	3 (8.8%)	0 (0%)	1 (4.5%)
***RET*** **fusions**	1 (2.9%)	0 (0%)	0 (0%)

*Abbreviations*: *LUAD* lung adenocarcinoma, *LUSC* lung squamous cell carcinoma, *NSCLC* non-small cell lung cancer, *N* number, *NOS* not otherwise specified

**Table 2:** Genomic characteristics of patients with oncogene-driven mutations on TKI treatment

Patient ID	Age	Gender	Ethnicity	Driver Oncogene Mutation	PD-L1 IHC	TMB	TKI Treatment	Clinical response	RECIST V1.1	PFS (mos)	OS (mos)
1	78	F	Asian	*EGFR* exon 19 deletion	0	NA	Erlotinib, Osimertinib	Good	PR	16.2	32.8
2	72	F	Asian	*EGFR* L858R, *EGFR* T790M	2%	4	Erlotinib, Afatinib, Osimertinib	Poor	PD	7.7	46.6
3	76	F	Asian	*EGFR* L858R	NA	NA	Gefitinib, Afatinib	Good	PR	7	25.3
4	73	F	NHW	*EGFR* exon 19 deletion	0	4	Afatinib, Osimertinib	Good	PR	16	19.9
5	79	F	NHW	*ERBB2* L755	0	7	Afatinib	Good	PR	23.9	38.4
6	43	M	Asian	*EGFR* exon 19 deletion	50%	4	Osimertinib	Poor	PD	0.7	7.8
7	69	M	NHW	*EGFR* L858R, *EGFR* T790M	30%	6	Osimertinib	Poor	PD	4.5	14.5
8	76	F	H	*EGFR* L858R, *EGFR* T790M, C797S	5%	5	Afatinib, Brigatinib	Poor	PD	2	3.9
9	57	F	Asian	*EGFR* L858R, *EGFR* T790M	0	8	Osimertinib	Poor	PR	20.4	21.5
10	85	F	Asian	*EGFR* L858R, *EGFR* T790M	0	9	Osimertinib	Poor	PR	4.4	6.5
11	55	M	Asian	*EGFR* exon 19 deletion	0	7.4	Osimertinib	Good	SD	21	33
12	68	F	NHW	*EGFR* exon 19 deletion	70%	7	Osimertinib	Poor	SD	0.9	7.9
13	67	F	NHW	*EGFR* L858R	100%	16	Osimertinib	Poor	SD	6	6
14	79	M	Asian	*EGFR* L858R	0	2	Erlotinib	Good	PR	10.5	26.2
15	79	M	Asian	*EGFR* L858R, *EGFR* T790M	0	2	Osimertinib	Good	PR	105	26.2
16	53	F	Asian	*EGFR* exon 19 deletion	50%	4	Erlotinib	Good	PR	25.1	49
17	82	F	Asian	*METTL25-ALK* fusion	15%	2	Alectinib	Good	PR	37	37
18	73	M	Asian	*MET* amplification	30%	14	Crizotinib	Good	PR	10.6	10.7
19	45	M	Asian	*EML4-ALK* V3a/b fusion	50%	6	Alectinib	Good	PR	16	38.4
20	64	M	NHW	*EGFR* exon 19 deletion	1%	4	Osimertinib	Good	PR	12.6	21.3
21	58	M	Asian	*EML4-ALK* V4a/b fusion	50%	0	Alectinib	Good	PR	60.6	60.6
22	60	F	Asian	*KIF5b-RET* fusion	80%	0	Alectinib	Poor	PD	6.2	14.1
23	79	F	NHW	*EGFR* exon 20 insertion	10%	7.4	Poziotinib	Good	PD	8.7	18.6
24	72	F	NHW	*EGFR* exon 20 insertion	0	3.7	Osimertinib	Poor	PD	3.7	3.7
25	56	F	Asian	*EGFR* exon 19 deletion	> 1%	6.3	Osimertinib	Poor	PD	2.1	2.1
26	59	F	NHW	*EGFR* L858R	8%	2.5	Osimertinib	Poor	SD	10	15.9
27	51	F	Asian	*EGFR* exon 19 deletion	6%	11.6	Osimertinib	Poor	PD	3.9	3.9
28	61	F	NHW	*CCDC6-RET* fusion	0	2.6	Selpercatinib	Good	SD	5.8	5.8
29	59	M	NHW	*HER2* amplification	0	9.5	Afatinib	Good	SD	8	8
30	69	F	NHW	*MET* exon 14 mutation	25%	11	Campactinib	Good	PR	1.9	1.9
31	66	F	NHW	*CD47-MET* fusion; *MET* missense mutation	0	1.1	Campactinib	Good	PR	1.5	1.5
32	73	M	NHW	*EGFR* exon 19 deletion	0	1	Osimertinib	Good	PR	6.3	9.5
33	74	M	NHW	*ERBB2* exon 20 insertion	0	3.2	Poziotinib	Poor	PD	1.6	18.1
34	79	F	NHW	*EGFR* Exon 20 insertion	10%	7.4	Osimertinib	Poor	PD	4.1	18.6

**Table 3:** Comparisons of baseline blood biomarker levels between different NSCLC cohorts

Baseline Biomarker	Cohort A		Cohort B		Cohort C		***P***-value^**$**^
	N	Mean (±SD)	N	Mean (±SD)	N	Mean (±SD)	
**WBC (×10**^**3**^**cells/μL)**	27	8.4 (±3.7)	35	7.5 (±2.5)	22	9.2 (±5.3)	0.750
**Hemoglobulin (gram/dL)**	27	12.5 (±2.0)	35	12.7 (±1.9)	22	12.8 (±1.4)	0.805
**Platelet count (× 10**^**3**^**cells/**μL**)**	27	305.2 (±126.6)	35	298.8 (±125.9)	21^a^	281.0 (±89.8)	0.884
**ANC (×10**^**3**^**cells/**μL**)**	27	6.1 (±3.4)	35	5.6 (±2.1)	22	6.9 (±5.1)	0.948
**ALCs (×10**^**3**^**cells/μL)**	27	1.3 (±0.8)	35	1.1 (±0.6)	22	1.2 (±0.8)	0.571
**CD3 + CD4 + %**	27	40.6 (±11.1)	35	45.3 (±12.7)	22	44.5 (±9.5)	0.222
**CD3 + CD8 + %**	27	23.9 (±9.6)	35	27.1 (±12.5)	22	26.4 (±9.5)	0.427
**CD3 + %**	27	**65.7 (±10.3)**	35	72.4 (±10.3)	22	72.3 (±8.8)	**0.014**
**CD3 + CD4+ count (cells/μL)**	27	512.9 (±282.9)	35	492.3 (±288.1)	22	598.7 (±402.9)	0.793
**CD3 + CD8+ count (cells/μL)**	27	318.3 (±242.3)	35	305.5 (±260.1)	22	326.7 (±189.0)	0.705
**CD4/CD8 ratio**	27	2.0 (±0.9)	35	2.1 (±1.3)	22	1.9 (±0.9)	0.934
**CD4 plus CD8 count (cells/μL)**	27	831.2 (±451.7)	35	797.8 (±431.7)	22	925.4 (±555.5)	0.831
**dNLR**	27	3.4 (±3.0)	35	3.2 (±1.5)	22	3.4 (±2.5)	0.668

^$^*P*-values from Kruskal-Wallis tests. Bold for statistical significance

^a^One patient had clumped platelets

### Cancer treatment modulated blood immune cells in NSCLC patients

**Fig. 3:**
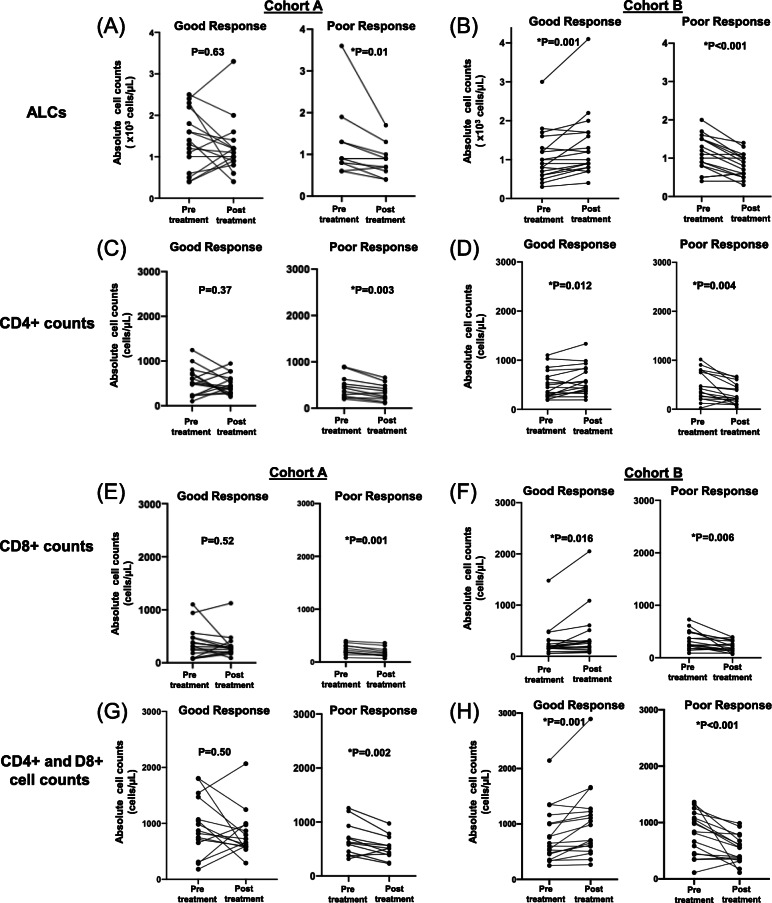
Comparison of changes in ALCs, CD4, CD8 and CD4 plus CD8 cell counts pre- and after-treatment. ALCs, CD4, CD8, and CD4 plus CD8 counts by flow cytometry in good responders and poor responders in NSCLC patients receiving TKI treatment (Cohort A) (**A**, **C**, **E**, **G**) or receiving ICI (Cohort B) (**B**, **D**, **F**, **H**) are shown. Groups were compared by the Wilcoxon signed rank test. *P < 0.05 for statistical significance. Abbreviations: ALCs, absolute lymphocyte cells; ICI, immune checkpoint inhibitors

Table 4 and Table 5 summarize the changes of 13 blood cell types in PBMCs in patients with mNSCLC with (Cohort A) or without (Cohort B) driver oncogenes, respectively, who had paired pre- and post-treatment blood samples. In cohort A (*N* = 27), TKI treatment was associated with significant decreases in white blood cells (WBCs, − 3.5 ± 1.1 × 10^3^ cells/μL, *P* = 0.015), absolute neutrophil counts (ANCs, − 3.6 ± 1.1 cells/μL, *P* = 0.014), and dNLR (− 2.4 ± 1.2, *P* = 0.027) but increased CD3% (6.4 ± 2.5%, *P* = 0.041) in poor responders versus good responders (Table 4). Figure 3 illustrates the major changes in ALCs, CD4, CD8, and CD4 plus CD8 cell counts in good responders (left panel) and poor response (right panel), respectively. In cohort A, the patients with oncogene-driven NSCLC received targeted therapy with specific TKI. Flow cytometry analysis showed that TKI treatment had various effects on the blood immune cells (Fig. 3a, c, e, g). Of 16 patients with good clinical response to TKI therapy (left panel), 10 (62.5%) patients had decreased ALCs and 6 (37.5%) patients had increased ALCs (Fig. 3a, left). Similar changes were observed in post-treatment CD4 counts (Fig. 3c, left), CD8 counts (Fig. 3e, left), and CD4 plus CD8 counts (Fig. 3g, left), respectively, in patients with good clinical responses to TKI treatment. In contrast, all 11 patients with poor clinical response to TKIs had significantly decrease in ALCs (from 1200 ± 900 to 900 ± 400 × 10^3^ cells/μL, *P* = 0.01) (Fig. 3a, right), the CD4 cell counts (from 472 ± 245 to 345 ± 180 cells/μL, *P* = 0.003) (Fig. 3c, right), the CD8 cell counts (from 240 ± 98 to 184 ± 89 cells/μL, *P* = 0.001) (Fig. 3e, right), and the CD4 plus CD8 cell counts (from 711 ± 301 to 529 ± 227 cells/μL, *P* = 0.002) (Fig. 3g, right), respectively. These results suggested that small molecule TKIs may modulate blood immune cell count in patients with oncogene-driven NSCLC.

**Table 4:** Comparisons of baseline/post/change between good vs poor responders in Cohort A

Response	Good Response			Poor Response			Difference (Good - Poor)					
Timepoints	Baseline	Post-treatment	Change (Post - Pre)	Baseline	Post-treatment	Change (Post - Pre)	Baseline		Post-treatment		Change	
**Blood biomarker**	**Mean (±SD)**	**Mean (±SD)**	**Mean (±SD)**	**Mean (±SD)**	**Mean (±SD)**	**Mean (±SD)**	**Mean (±SE)**	**P-value**^**$**^	**Mean (±SE)**	**P-value**^**$**^	**Mean (±SE)**	**P-value**^**$**^
**WBC (×10**^**3**^**cells/μL)**	9.2 (±3.7)	6.3 (±2.3)	−2.9 (±3.1)	7.2 (±3.4)	7.9 (±3.5)	0.6 (±2.6)	1.9 (±1.4)	0.092	− 1.6 (±1.1)	0.309	**−3.5 (±1.1)**	**0.015**
**Hemoglobulin (gram/dL)**	12.6 (±2.2)	12.6 (±1.8)	0.0 (±1.8)	12.2 (±1.7)	11.8 (±1.8)	−0.4 (±0.8)	0.4 (±0.8)	0.480	0.8 (±0.7)	0.210	0.3 (±0.6)	0.807
**Platelet count (×10**^**3**^**cells/μL)**	339.9 (±121.6)	248.7 (±96.1)	−91.3 (±103.8)	254.7 (±121.5)	234.5 (±96.5)	−20.2 (±95.9)	85.2 (±47.6)	0.092	14.1 (±37.7)	0.643	−71.1 (±39.5)	0.079
**ANC (×10**^**3**^**cells/μL)**	6.8 (±3.6)	4.4 (±2.3)	−2.5 (±3.2)	5.1 (±3.0)	6.2 (±3.3)	1.1 (±2.5)	1.7 (±1.3)	0.237	−1.9 (±1.1)	0.095	**−3.6 (±1.1)**	**0.014**
**ALCs (×10**^**3**^**cells/μL)**	1.4 (±0.7)	1.2 (±0.7)	−0.2 (±0.7)	1.2 (±0.9)	0.9 (±0.4)	−0.4 (±0.5)	0.2 (±0.3)	0.395	0.4 (±0.2)	0.078	0.2 (±0.3)	0.575
**CD3 + CD4 + %**	38.7 (±10.9)	39.4 (±8.8)	0.7 (±8.0)	43.3 (±11.2)	39.0 (±9.0)	−4.3 (±4.5)	−4.6 (±4.3)	0.276	0.4 (±3.5)	0.922	5.0 (±2.7)	0.100
**CD3 + CD8 + %**	23.9 (±9.3)	24.8 (±7.8)	0.9 (±3.9)	23.8 (±10.4)	23.2 (±10.9)	−0.6 (±2.2)	0.1 (±3.8)	0.806	1.6 (±3.6)	0.436	1.5 (±1.3)	0.234
**CD3 + %**	64.3 (±9.0)	66.0 (±8.0)	1.7 (±7.0)	67.8 (±12.0)	63.1 (±10.0)	−4.7 (±5.5)	−3.5 (±4.0)	0.357	2.9 (±3.5)	0.355	**6.4 (±2.5)**	**0.041**
**CD3 + CD4+ count (cells/μL)**	541.4 (±311.1)	466.3 (±213.0)	−75.1 (±314.4)	471.5 (±244.6)	344.9 (±179.5)	−126.5 (±94.8)	69.9 (±112.1)	0.592	121.4 (±78.4)	0.157	51.5 (±98.2)	0.592
**CD3 + CD8+ count (cells/μL)**	372.3 (±296.3)	307.7 (±232.8)	−64.6 (±234.0)	239.9 (±97.6)	184.1 (±88.6)	−55.8 (±30.2)	132.3 (±93.1)	0.321	123.6 (±74.0)	0.041	−8.7 (±71.4)	0.714
**CD4/CD8 ratio**	1.9 (±0.8)	1.8 (±0.8)	−0.1 (±0.6)	2.1 (±1.0)	2.1 (±1.2)	0.0 (±0.5)	−0.3 (±0.3)	0.527	−0.3 (±0.4)	0.527	−0.1 (±0.2)	0.788
**CD4 plus CD8 count (cells/μL)**	913.6 (±524.8)	774.0 (±407.0)	−139.6 (±507.4)	711.4 (±301.2)	529.0 (±227.2)	− 182.4 (±119.1)	202.3 (±175.8)	0.321	245.0 (±135.7)	0.039	42.7 (±156.7)	0.643
**dNLR**	3.8 (±3.8)	2.6 (±1.8)	−1.2 (±3.8)	2.8 (±1.0)	4.0 (±2.0)	1.2 (±1.6)	1.00 (±1.2)	0.575	−1.4 (±0.7)	0.033	**−2.4 (±1.2)**	**0.027**

*Abbreviation*: *SD* Standard Deviation, *SE* Standard Error

^$^*P*-values from Wilcoxon rank sum tests. Bold for statistical significance

**Table 5:** Comparisons of baseline/post/change between good vs poor response in Cohort B

Response	Good Response			Poor Response			Difference (Good - Poor)					
Timepoints	Baseline	Post-treatment	Change (Post - Pre)	Baseline	Post-treatment	Change (Post - Pre)	Baseline		Post-treatment		Change	
Blood biomarker	Mean (±SD)	Mean (±SD)	Mean (±SD)	Mean (±SD)	Mean (±SD)	Mean (±SD)	Mean (±SE)	***P***-value^**$**^	Mean (±SE)	***P***-value^**$**^	Mean (±SE)	***P***-value^**$**^
**WBC (×10**^**3**^**cells/μL)**	7.1 (±2.5)	6.9 (±1.9)	−0.3 (±2.1)	8.0 (±2.4)	7.4 (±3.0)	−0.6 (±3.3)	−0.8 (±0.8)	0.329	−0.5 (±0.8)	0.756	0.3 (±0.9)	0.682
**Hemoglobulin (gram/dL)**	13.0 (±1.4)	12.6 (±1.3)	−0.4 (±1.5)	12.4 (±2.4)	11.7 (±1.6)	−0.7 (±2.8)	0.6 (±0.6)	0.388	0.9 (±0.5)	0.138	0.3 (±0.8)	0.434
**Platelet count (×10**^**3**^**cells/μL)**	278.2 (±98.3)	278.0 (±118.1)	−0.2 (±68.1)	300.0 (±152.1)	260.1 (±160.2)	−39.9 (±105.8)	−21.8 (±43.0)	0.961	17.9 (±47.4)	0.379	39.7 (±29.9)	0.321
**ANC (×10**^**3**^**cells/μL)**	5.2 (±2.3)	4.5 (±1.7)	−0.7 (±2.1)	6.0 (±1.9)	5.4 (±2.3)	−0.6 (±2.7)	−0.7 (±0.7)	0.249	−0.9 (±0.7)	0.230	−0.1 (±0.8)	0.844
**ALCs (×10**^**3**^**cells/μL)**	1.1 (±0.6)	1.3 (±0.8)	0.3 (±0.4)	1.1 (±0.5)	0.8 (±0.3)	−0.3 (±0.3)	−0.1 (±0.2)	0.523	0.5 (±0.2)	0.032	**0.6 (±0.1)**	**< 0.001**
**CD3 + CD4 + %**	48.8 (±12.6)	47.2 (±13.1)	−1.6 (±8.3)	41.6 (±11.9)	39.1 (±14.0)	−2.5 (±5.6)	7.2 (±4.2)	0.083	8.1 (±4.6)	0.111	0.9 (±2.4)	0.211
**CD3 + CD8 + %**	23.3 (±10.4)	25.4 (±12.6)	2.1 (±7.3)	31.0 (±13.5)	32.0 (±15.7)	−1.0 (±8.0)	−7.7 (±4.1)	0.098	−6.6 (±4.8)	0.200	1.1 (±2.6)	0.534
**CD3 + %**	72.0 (±7.8)	72.6 (±9.3)	0.6 (±8.1)	72.8 (±12.6)	71.5 (±13.4)	−1.3 (±5.3)	−0.8 (±3.5)	0.806	1.1 (±3.9)	0.961	1.8 (±2.3)	0.150
**CD3 + CD4+ count (cells/μL)**	502.9 (±284.4)	593.4 (±297.3)	90.6 (±136.1)	481.1 (±300.4)	310.8 (±204.2)	−170.2 (±209.4)	21.8 (±98.8)	0.831	282.6 (±86.7)	0.008	**260.8 (±59.3)**	**< 0.001**
**CD3 + CD8+ count (cells/μL)**	279.5 (±322.5)	400.1 (±476.1)	120.6 (±191.6)	333.1 (±178.1)	215.2 (±106.2)	− 117.9 (±153.2)	−53.6 (±88.8)	0.060	184.9 (±118.2)	0.321	**238.5 (±58.9)**	**< 0.001**
**CD4/CD8 ratio**	2.6 (±1.3)	2.4 (±1.3)	−0.2 (±0.9)	1.6 (±1.2)	1.7 (±1.2)	0.0 (±0.7)	1.0 (±0.4)	0.033	0.7 (±0.4)	0.089	−0.2 (±0.3)	0.922
**CD4 plus CD8 count (cells/μL)**	782.4 (±482.8)	993.6 (±627.4)	211.2 (±268.1)	814.2 (±384.3)	526.1 (±251.2)	−288.1 (±329.2)	−31.8 (±148.1)	0.567	467.5 (±163.4)	0.011	**499.3 (±101.2)**	**< 0.001**
**dNLR**	3.1 (±1.7)	2.4 (±1.8)	−0.7 (±1.6)	3.4 (±1.3)	3.9 (±2.7)	0.6 (±2.5)	−0.2 (±0.5)	0.590	−1.5 (±0.8)	0.030	−1.3 (±0.7)	0.160

^$^*P*-values from Wilcoxon rank sum tests. Bold for statistical significance

*Abbreviation*: *SD* Standard Deviation, *SE* Standard Error

In cohort B (*N* = 35), the patients without oncogene-driven NSCLC received a PD-(L)1 ICI (i.e., pembrolizumab, nivolumab, or atezolizumab), either alone (*N* = 14) or in combination with chemotherapy (*N* = 21) (Table 5). Compared to pre-treatment, ICI treatment was associated with significant increases in post-treatment ALCs (from 1100 ± 600 to 1300 ± 800 × 10^3^ cells/μL, *P* < 0.001) (Fig. 3b, left), CD4 counts (from 503 ± 284 to 593 ± 297 cells/μL, *P* = 0.004) (Fig. 3d, left), CD8 counts (from 280 ± 323 to 400 ± 476 cells/μL, *P* = 0.006) (Fig. 3f, left), and CD4 plus CD8 cell counts (from 782 ± 483 to 994 ± 627 cells/μL, *P* < 0.001) (Fig. 3h, left), respectively, in good responders (*N* = 18). In contrast, ICI treatment was associated with significant decreases in post-treatment in ALCs (from 1100 ± 500 to 800 ± 300 × 10^3^ cells/μL, *P* < 0.001) (Fig. 3b, right), CD4 counts (from 481 ± 300 to 311 ± 204 cells/μL, *P* = 0.004) (Fig. 3d, right), CD8 counts (from 333 ± 178 to 215 ± 106 cells/μL, *P* = 0.006) (Fig. 3f, right), and CD4 plus CD8 counts (from 814 ± 384 to 526 ± 251 cells/μL, *P* < 0.001) (Fig. 3h, right), respectively, in poor responders to ICI (*n* = 17). There results were comparable to patients with oncogene-driven NSCLC who had poor clinical response to TKI (Fig. 3, cohort A, right).

### Post-treatment lymphopenia was associated with poor clinical benefit in NSCLC patients

**Fig. 4:**
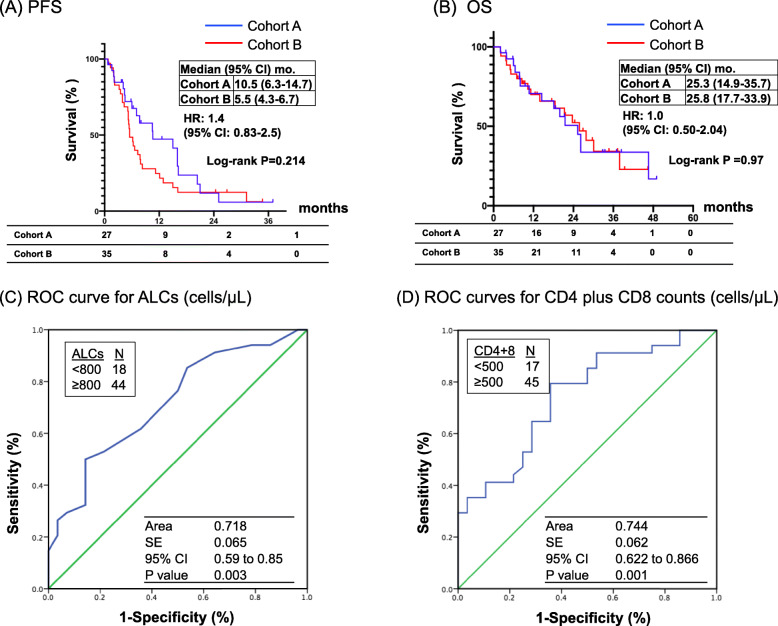
Kaplan-Meier PFS and OS estimates by lymphocyte counts according to NSCLC cohorts. Median PFS (**A**) and OS (**B**) of NSCLC patients are illustrated according to cohort A and B, respectively. Groups were compared using the log-rank test. Receiver operating characteristic (ROC) curves analysis for ALCs (**C**) and CD4 plus CD8 (**D**) in enrolled NSCLC patients (*N* = 62) are shown. Tick marks indicate censored data. Groups were compared using the Z test. *P* < 0.05 for statistical significance

**Fig. 5:**
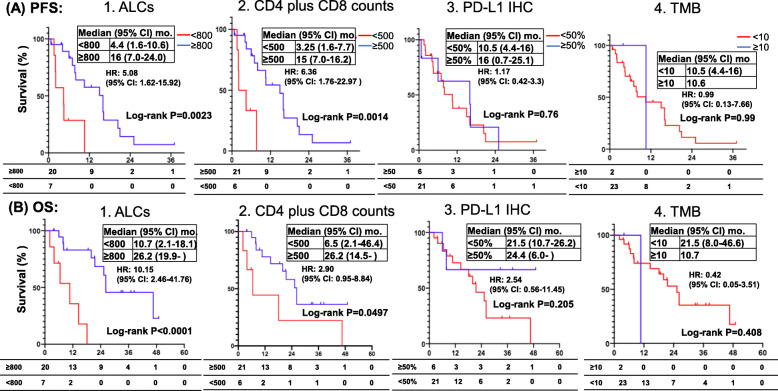
Kaplan-Meier PFS and OS estimates according to ALCs, CD4 plus CD8 counts, PD-L1 IHC and TMB in cohort A. Post-treatment low ALCs (< 800 cells/μL, red) were associated with shorter PFS and OS compared to high ALCs (≥800 cells/μL, blue) in patients with oncogene-driven NSCLC (cohort A) (**A** and **B**), respectively. Post-treatment low CD4 plus CD8 cell counts (< 500 cells/μL, red) were associated with shorter PFS and OS compared to high CD4 plus CD8 cell counts (≥500 cells/μL, blue) (cohort A) (**A** and **B**), respectively. PD-L1 IHC expression (**C**) and TMB status (**D**) did not affect the PFS and OS in NSCLC patients with oncogene driven NSCLC. Tick marks indicate censored data. Groups were compared using the log-rank test. *P* < 0.05 for statistical significance

With a median follow-up of 24.5 months, there were no significant differences in median PFS (10.6 vs. 5.5 months, *P* = 0.20) and OS (25.3 vs. 25.8 months, *P* = 0.94) in cohort A and cohort B (Fig. 4). The correlation of the post-treatment ALCs, CD4 plus CD8 counts, PD-L1 IHC, and TMB were determined with the clinical outcomes in each cohort of NSCLC patients. Currently, there is no established cutoff for ALCs as a biomarker [19–22]. Using the receiver operating characteristic (ROC) curve and Youden index analysis, we identified 800 and 500 cells/μl as the optimal cut-off values for ALCs and CD4+ plus CD8+ cell counts, respectively (Fig. 4c). Figure 5 illustrates the median PFS and OS according to ALCs, CD4 plus CD8 counts, PD-L1 IHC, and TMB in cohort A patients (*N* = 27). Compared to those patients with post-TKI treatment ALCs < 800 cells/μL, patients with post-TKI treatment ALCs ≥800 cells/μL had longer median PFS (16.0 vs. 4.4 months; HR 5.08, 95% CI 1.62–15.92, *P* = 0.0023) (Fig. 5A1) and longer median OS (26.2 vs. 10.7 months; HR 10.15, 95% CI 2.46–41.76, *P* < 0.0001) (Fig. 5B1), respectively. Similarly, patients with post-TKI treatment CD4 plus CD8 counts ≥500 cells/μL had statistically better PFS and OS compared to those patients with CD4 plus CD8 counts < 500 cells/μL (Fig. 5A2 and B2). In contrast, PD-L1 IHC and TMB expression in patients with oncogene-driven NSCLC did not correlate with the PFS (Fig.5A3 and A4) and OS (Fig. 5B3 and 5B4). Multivariate analysis using the Cox proportional hazards regression model showed ALCs remained significant for PFS (*P* = 0.024, HR 4.25, 95% CI 1.2–14.9) and OS (*P* = 0.022, HR 7.59, 95% CI 1.33–43.16), while CD4 plus CD8 counts was only significant for PFS (*P* = 0.006, HR 8.89, 95% CI 1.89–41.86) in cohort A (Tables 6 and 7).

**Table 6:** Univariable and multivariable survival analysis for OS using Cox proportional hazards models

Group	Immune Biomarker	Univariable		Multivariable	
		HR (95% CI)	***p***-value	HR (95% CI)	***p***-value
**Cohort A**	ALCs > = 800 < 800	10.15 (2.46, 41.76)	0.001	7.59 (1.33, 43.16)	0.022
	CD4 plus CD8 counts > = 500 < 500	2.90 (0.95, 8.84)	0.061	–	–
	PD-L1 IHC > = 50% < 50%	2.54 (0.56, 11.45)	0.226	–	–
	TMB > = 10 < 10	0.42 (0.05, 3.51)	0.423	–	–
**Cohort B**	ALCs > = 800 < 800	2.38 (0.89, 6.35)	0.084	–	–
	CD4 plus CD8 counts > = 500 < 500	3.03 (1.12, 8.20)	0.029	5.96 (1.60, 22.20)	0.008
	PD-L1 IHC > = 50% < 50%	3.18 (0.97, 10.45)	0.056	–	–
	TMB > = 10 < 10	2.18 (0.71, 6.75)	0.175	–	–

**Table 7:** Univariable and multivariable survival analysis for PFS using Cox proportional hazards models

Group	Immune Biomarker	Univariable		Multivariable	
		HR (95% CI)	***p***-value	HR (95% CI)	***p***-value
**Cohort A**	ALCs > = 800 < 800	5.08 (1.62, 15.92)	0.005	4.25 (1.21, 14.87)	0.024
	CD4 plus CD8 counts > = 500 < 500	6.36 (1.76, 22.97)	0.005	8.89 (1.89, 41.86)	0.006
	PD-L1 IHC > = 50% < 50%	1.17 (0.42, 3.30)	0.764	–	–
	TMB > = 10 < 10	0.99 (0.13, 7.66)	0.989	–	–
**Cohort B**	ALCs > = 800 < 800	2.63 (1.16, 5.99)	0.021	2.50 (1.05, 5.95)	0.038
	CD4 plus CD8 counts > = 500 < 500	2.70 (1.21, 6.04)	0.016	2.94 (1.18, 7.37)	0.021
	PD-L1 IHC > = 50% < 50%	2.36 (1.04, 5.37)	0.040	5.21 (1.77, 15.38)	0.003
	TMB > = 10 < 10	3.16 (1.27, 7.86)	0.013	4.11 (1.58, 10.70)	0.004

**Fig. 6:**
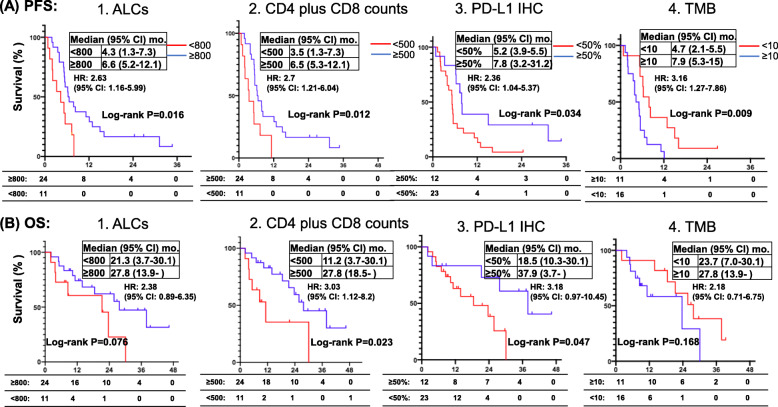
Kaplan-Meier PFS and OS estimates according to ALCs, CD4 plus CD8 counts, PD-L1 IHC and TMB in cohort B. In patients without oncogene-driven NSCLC (cohort B), post-treatment low ALCs (< 800 cells/μL, red) were associated with statistically significant shorter PFS (**A**) but not OS (**B**) compared to high ALCs (≥800 cells/μL, blue). Post-treatment low CD4 plus CD8 cell counts (< 500 cells/μL, red) were associated with shorter PFS and OS compared to high CD4 plus CD8 cell counts (≥500 cells/μL, blue) (cohort B) (**A** and **B**), respectively. Patients with NSCLC expressing PD-L1 IHC < 50% had shorter PFS and shorter OS compared to those patients with NSCLC expressing PD-L1 IHC ≥50% (**C**). Patients with NSCLC expressing TMB ≥10 Mut/Mb had shorter PFS but not OS compared to those patients with NSCLC expressing TMB < 10 Mut/Mb (**D**). Tick marks indicate censored data. Groups were compared using the log-rank test. *P* < 0.05 for statistical significance

Figure 6 illustrates the median PFS and OS according to ALCs, CD4 plus CD8 counts, PD-L1 IHC, and TMB in cohort B patients (*N* = 35). Patients without oncogene-driven NSCLC whose post-ICI ALCs ≥800 cells/μL had significantly longer median PFS (6.6 vs. 4.3 months, HR 2.63, 95% CI 1.16–5.99, *P* = 0.016) (Fig. 6A1) but not median OS benefit (27.8 vs. 21.3 months, HR 2.38, 95% CI 0.89–6.35, *P* = 0.076) (Fig. 6B1). Patients with CD4 plus CD8 counts ≥500 cells/μL had significantly longer median PFS (6.5 vs 3.5 months, HR 2.7, 95% CI: 1.21–6.04, *P* = 0.012) (Fig. 6A2) and longer median OS (27.8 vs. 11.2 months, HR 3.03, 95% CI 1.12–8.2, *P* = 0.026) (Fig. 6B2). Patients with PD-L1 IHC ≥50% had significantly longer median PFS (7.8 vs. 5.2 months, HR 2.36, 95% CI: 1.04–5.37, *P* = 0.034) (Fig. 6A3) and longer median OS (37.9 vs. 18.5 months, HR 3.18, 95% CI 0.97–10.45, *P* = 0.047) (Fig. 6B3). Patients with TMB expression ≥10 mut/mb had significantly longer median PFS (7.9 vs. 4.7 months, HR 3.16, 95% CI: 1.27–7.86, *P* = 0.009) (Fig. 6A4). In contrast, TMB expression did not correlate with OS (Fig. 6B4) in patients without oncogene-driven NSCLC. Multivariate analysis using the Cox proportional hazards regression model revealed that ALCs, CD4 plus CD8 counts, PD-L1 IHC and TMB are an independent significant predictor for PFS (*P* = 0.038, *P* = 0.021, *P* = 0.003 and *P* = 0.004, respectively), and CD4 plus CD8 counts remained a significant predictor for OS (*P* = 0.008, HR 5.96, 95% CI 1.60–22.2), for NSCLC patients without driver oncogenes receiving ICI treatment (Tables 6 and 7).

### Immunophenotypic changes of PBMCs in a patient who received ICI combination

**Fig. 7:**
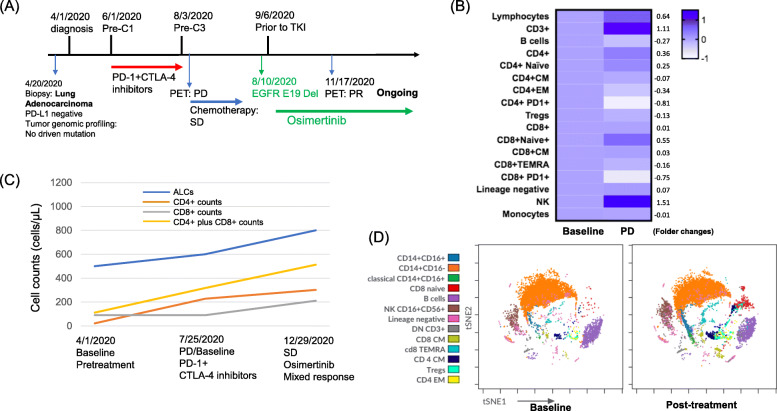
Immunophenotypic changes of PBMCs in a patient who received ICI combination. **A** summarizes the key events in the clinical course of a patient with EGFR exon 19 deletion. A 72-year-old gentleman, never smoker was diagnosed with cT4N2M1c NSCLC adenocarcinoma with large tumor burden in April 2020 when he presented with worsening non-productive cough and shortness of breath for about 6 months. The tumor was stained negative for PD-L1 IHC stain (DAKO, clone 22C3). Tumor genomic profiling did not identify any driver oncogenes. The patient received PD-1 and CTLA-4 inhibitors for 3 cycles and was found to have tumor progression. The patient subsequently received carboplatin and pemetrexed for two cycles without significant clinical improvement. Repeat tumor genomic profiling test identified an EGFR E19Del. The patient had been in clinical PR to EGFR TKI osimertinib for over 10 months at the time of this report. The changes in ALCs, CD4, CD8, and CD4 plus CD8 cell counts during the treatment course (**B**). Heat mapping (**C**) and viSNE land (**D**) illustrate the immunophenotypic changes of PBMCs before and after ICI treatment

Figure 7a summarizes the clinical course of a patient with *EGFR* exon 19 deletion who received ICIs at diagnosis when tumor genomic profiling test did not identify the *EGFR* mutation. Patient subsequently received platinum-based chemotherapy for 2 cycles and has been having clinical PR to EGFR TKI osimertinib. The changes in ALCs, CD4, CD8, and CD4 plus CD8 cell counts during the treatment course were shown in Fig. 7c. Although CD4+ and CD8+ T cells account for the majority of ALCs, ALCs include a few rare yet important lymphocyte subtypes such as natural killer (NK) cells and monocytes. In addition, changes in these lymphocytes might affect other immune cell types such as B cells in the PBMCs. Further, the immunophenotypic analysis of PBMCs collected before and after ICI treatment were performed for extended immune cell types, including B cells, NK cells, monocytes, using a multicolor flowcytometry analysis. As showed in heatmap (Figs. 7b) and viSNE land (Figs. 7d), the ICI treatment increased lymphocytes, CD3+, CD4+, CD4 TEMRA, CD8+, CD8 central memory, CD8 naïve cells and NK cells and decreased all other types of immune subtype cells including PD1 + CD4 and PD1 + CD8 cells.

### Patients with oncogene-driven NSCLCs have various responses to ICIs

**Fig. 8:**
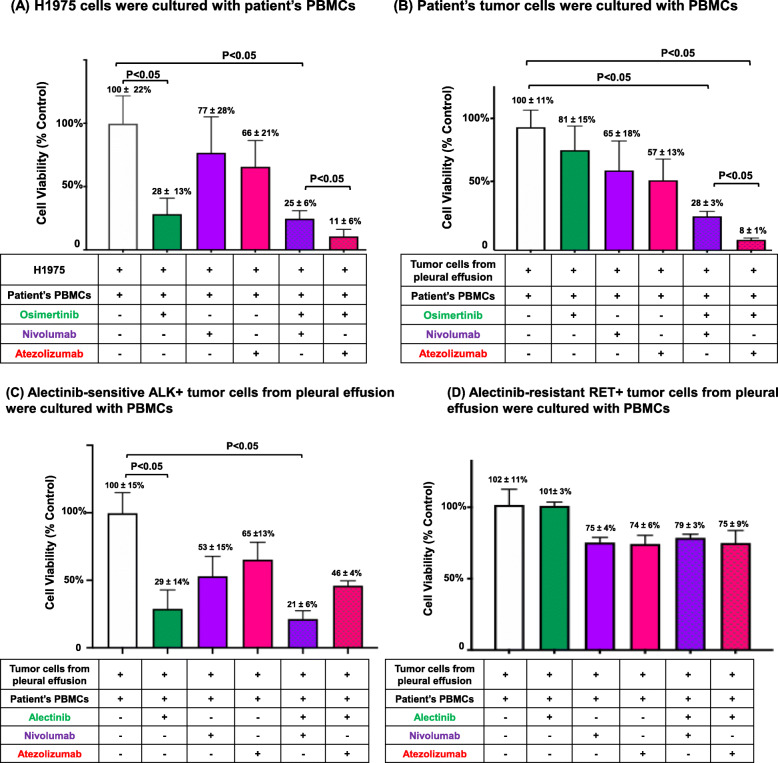
Growth inhibition of malignant tumor cells and PBMCs from the same patients with oncogene-driven NSCLC by TKIs and ICIs. Tumor cells isolated from malignant pleural effusion and PBMCs isolated from the peripheral blood of individual patients were cocultured for 12 h before treated with specific TKI and/or ICIs. H1975 cells (**A**) and osimertinib-resistant NSCLC cells (**B**) were cultured with patient’s PBMCs, and treated with vehicle, osimertinib (0.1 μM), nivolumab (10 μg/ml), atezolizumab (10 μg/ml) or combination as indicated for 72 h. Growth inhibition was measured using the MTS assay using vehicle as 100% control. Alectinib-sensitive ALK-fusion NSCLC cells (**C**) and alectinib-resistant RET-fusion NSCLC cells (**D**) were cocultured with PBMCs from corresponding patients and treated with vehicle, alectinib (0.1 μM), nivolumab (10 μg/ml), atezolizumab (10 μg/ml) or combination as indicated for 72 h. Growth inhibition was measured using the MTS assay with vehicle as 100% control. All data are shown as mean of triplicate samples. Error bars indicate standard deviation (SD). Groups were compared by the Wilcoxon signed rank test. *P* < 0.05 was considered statistically significant. Abbreviations: MTS, 3-(4,5-dimethylthiazol-2-yl)-5-(3-carboxymethoxyphenyl)-2-(4-sulfophenyl)-2H-tetrazolium)

We developed an in vitro cytotoxic assay using patient’s malignant pleural effusion and PBMCs to determine the effect of small molecule TKIs and ICIs on patient tumor cells and PBMCs (Fig. 8). As shown in Fig. 8a, osimertinib, nivolumab and atezolizumab inhibited the growth of H1975 cells by 28 ± 13%, 77 ± 28% and 66 ± 21%, respectively. In patient-derived, *EGFR*-mutant lung adenocarcinoma cells that were resistant to osimertinib (growth inhibition of 81 ± 15%), nivolumab and atezolizumab significantly inhibited the growth by (65 ± 18%) and (57 ± 13%), respectively, *P* < 0.05) (Fig. 8b). The addition of atezolizumab to osimertinib significantly inhibited the growth of both H1975 cells (11 ± 6%, *P* < 0.001) (Fig. 8a) and patient derived, *EGFR*-mutant NSCLC cells (8 ± 1%), (*P* < 0.001) (Fig. 8b). Osimertinb and atezolizumab had stronger cytotoxic synergism compared to osimertinib and nivolumab in both osimertinib-sensitive, *EGFR*-mutant (11 ± 6% vs 25 ± 6%, *P* < 0.05) (Fig. 8a) and osimertinib-resistant, *EGFR*-mutant lung adenocarcinomas (8 ± 1% vs 28 ± 3%, *P* < 0.05) (Fig. 8b).

ALK inhibitor alectinib, nivolumab and atezolizumab inhibited the growth of alectinib-sensitive, patient-derived *EML4-ALK* fusion NSCLC cells by 29 ± 14%, 53 ± 15%, 65 ± 13%, respectively. Although the addition of nivolumab to alectinib did not significantly increase the growth of tumor cells compared to alectinib or nivolumab alone (21 ± 6%, 29 ± 14%, vs 53 ± 15%, respectively), and the addition of atezolizumab to alectinib had antagonistic effect on tumor cell growth compared to alectinib alone (46 ± 4% vs 29 ± 14%, respectively) (Fig. 8c). In alectinib-resistant *RET* fusion NSCLC cells from another patient, nivolumab or atezolizumab, either alone or in combination with alectinib, did not significant inhibit the growth of tumor cells (102 ± 11%, 75 ± 4%, 74 ± 6%, 79 ± 3%, 75 ± 9% respectively) (Fig. 8d). These data support that patients with oncogene-driven NSCLCs could have various responses to ICIs either alone or in combination with TKIs. However, we could not verify the clinical response of ICI and ICI-TKI combination in patients as these were contraindicated clinically.

## Discussion

Our study has several clinical implications. First, lymphocyte counts and immunophenotyping of T-cell and B-cell have been used to assess the immune status and are prognostic biomarkers. Low levels of lymphocytes in the blood could indicate an increased risk for death [32]. The American Society of Hematology (ASH)-American Society for Transplantation and Cellular Therapy (ASTCT) defined patients with neutropenia ANC < 500 cells/μL and lymphopenia ALCs< 200 cells/μL as immunodeficiencies and recommended against COVID-19 vaccination [33]. We found that all study patients had ALCs > 200/μL during almost the entire disease course (except 3 patients at imminent dying stage), which is safe for receiving COVID-19 vaccination [34]. It is well known that many cancer treatments for hematological malignancies are immunosuppressive, which include cytotoxic chemotherapy, monoclonal antibodies against CD20, CD38 and CD52, calcineurin inhibitors, Mammalian Target of Rapamycin (mTOR), bortezomib and Bruton’s tyrosine kinase (BTK) inhibitors [35, 36]. A recent study showed that severe lymphopenia (ALCs < 500 cells/μL) before starting the consolidation durvalumab in patients with unresected locally advanced NSCLC after completion of definitive chemoradiation was associated with worse PFS compared to those patients without severe lymphopenia [37]. Although immune modulation is well documented in targeted therapy with monoclonal antibody for solid tumors [38], there is no prior study to determine the effect of TKIs on blood immune cells in patients with oncogene-driven NSCLC. In large clinical trials of TKIs for patients with advanced oncogene-driven NSCLC, the incidence and severity of leukopenia, neutropenia, and lymphopenia are generally less than 5% (Table 6) [39–49]. We observed that at baseline the patients with oncogene-driven NSCLC in cohort A had lower CD3% compared to patients without oncogene-driven NSCLC in cohort B and control group in cohort C. After TKI treatment, and these patients in cohort A had significantly increases in CD3% and decreased WBC, ANC and dLNR (Table 4). Consistent with previous reports, we also found that cancer progression [50] cytoreduction by chemotherapy and/or radiation [51–54], infection [32], and steroids use [55] were common reasons associated with decreased lymphocyte counts in patients with mNSCLC. This could reverse promptly with the clinical improvement or stopping steroids. While the changes of immune cell counts were associated with good or poor clinical responses to ICIs, targeted therapy with TKIs could modulate immune cell counts that mimic good or poor clinical responses to ICIs. The impact of our observation on patient’s response to ICIs is unknown and needs further exploration.

Second, small molecule TKIs targeting a growing number of *gain-of-function* molecular targets, such as *EGFR, BRAF V600E, MET* exon 14 skipping mutations, and *ALK-, ROS1-*, and neurotrophic receptor tyrosine kinase (*NTRK*)- or *RET*-gene fusions have been shown to improve PFS and OS with favorable toxicity profiles compared to platinum-containing combination chemotherapy as first-line systemic therapy in ~ 25% of NSCLC patients (Table 8). It is important to understand the role of TKIs on the immune system and treatment with ICIs. Compared to molecular biomarkers, immune biomarkers are complex with many different components that are subjected to change during natural tumor progression and treatment. A coordinated response by both humoral immunity and cell-mediated immunity is important to the response to ICIs [64, 65]. Using multiplex flow cytometry, we performed the phenotypic analysis of various immune cells in the patient PBMCs. These immune cells include T cell subsets (such as effector, activated, memory, exhausted, and regulatory), B cells, and NK/NKT cells. CD8+ T cells are the primary effector cells against tumors. The presence of activated, circulating, tumor-derived, PD1 + CD8+ T cells in patient PBMCs have been associated with clinical response to ICI therapy. The tumor-antigen specificities and TCR repertoires of the circulating and tumor-infiltrating CD8 + PD-1+ cells appeared similar. We observed that CD4 plus CD8 counts were an independent biomarker for PFS of NSCLC patients in both cohort A and B, and OS of NSCLC patients in cohort B. ALCs were an independent biomarker for OS in patients with oncogene-driven NSCLC. In cohort B, post-treatment CD4 plus CD8 cell counts performed better than the known immune biomarkers (PD-L1 IHC and TMB) in predicting response to ICIs. Further characterization of the effect of TKIs on the expression and function of immune cells in oncogene-driven NSCLC are warranted.

**Table 8:** Reported incidence of hematological adverse events in clinical trials of TKIs in NSCLC

Drug	Trial Name	ORR (CR/PR)	mPFS (mo)	Neutropenia		Lymphopenia		Anemia		Thrombocytopenia		Reference
				All grades (%)	Grade 3 and 4 (%)	All grades (%)	Grade 3 and 4 (%)	All grades (%)	Grade 3 and 4 (%)	All grades (%)	Grade 3 and 4 (%)	
**Osimertinib**	FLAURA (NCT02296125)	80% (3%/77%)	18.9	NA	4/279 (1.4%)	NA	4/279 (1.4%)	34/279 (12.2%)	3/279 (1.1%)	NA	2/279 (0.7%)	Soria JC (2018); Ramalingam SS (2020) [43, 44]
**Osimertinib**	ADAURA (NCT02511106)	NA	NA	NA	1/337 (0.3%)	NA	NA	NA	NA	NA	NA	Wu YL (2020) [46]
**Erlotinib and ramucirumab**	RELAY (NCT02411448)	76% (1%/75%)	19.4	25/221 (11.3%)	6/221 (2.7%)	NA	NA	22/221 (10%)	4/221 (1.8%)	31/221 (14%)	3/221 (1.4%)	Nakagawa K (2019) [56]
**Erlotinib**	RELAY (NCT02411448)	75% (1%/74%)	12.4	16/225 (7.1%)	2/225 (0.89%)	NA	NA	10/225 (4.4%)	1/225 (0.44%)	6/225 (2.7%)	0/225 (0%)	Nakagawa K (2019) [56]
**Gefitinib**	ARCHER 1050 (NCT01774721)	72% (2%/70%)	9.2	4/224 (1.8%)	1/224 (0.45%)	2/224 (0.89%)	1/224 (0.45%)	16/224 (7.1%)	5/224 (2.2%)	NA	NA	Wu YL (2017); Mok TS (2018) [57, 58]
**Afatinib**	LUX-Lung 6 (NCT01121393)	66.9% (1.2%/65.7%)	11	2/239 (0.84%)	1/239 (0.42%)	NA	NA	19/239 (7.9%)	1/239 (0.42%)	4/239 (1.7%)	0/239 (0%)	Wu YL (2014) [59]
**Dacomitinib**	ARCHER 1050 (NCT01774721)	75% (5%/70%)	14.7	5/227 (2.2%)	0/227 (0%)	2/227 (0.88%)	0/227 (0%)	22/227 (9.7%)	2/227 (0.88%)	NA	NA	Wu YL (2017); Mok TS (2018) [57, 58]
**Alectinib**	J-ALEX (JapicCTI-132,316)	92% (2%/89%)	NA	3/103 (2.9%)	2/103 (1.9%)	NA	NA	6/103 (5.8%)	1/103 (0.97%)	NA	NA	Hida T (2017) [60]
**Brigatinib**	ALTA-1 L (NCT02737501)	71% (4%/67%)	12	2/136 (1.5%)	0/136 (0%)	NA	NA	NA	NA	NA	NA	Camidge DR (2018) [61]
**Brigatinib**	ALTA (NCT02094573)	Arm B: 54%	12.9	NA	NA	NA	NA	NA	NA	NA	NA	Kim D (2021) [62]
**Poziotinib**	ZENITH20 (NCT03318939)	27.8%	5.5	NA	NA	NA	NA	NA	NA	NA	NA	Ternyila D (2020) [63]
**Capmatinib**	Geometry Mono-1 (NCT02414139)	41% (0%/41%)	5.4	NA	NA	NA	NA	NA	NA	NA	NA	Wolf J (2020) [48]
**Selpercatinib**	LIBRETTO-001 (NCT03157128)	64% (2%/62%)	16.5	NA	NA	NA	NA	NA	NA	NA	NA	Drilon A (2020) [49]

*Abbreviations CR* complete response, *mo* month, *mPFS* median progression free survival, *NA* not available, *ORR* overall response rate, *PR* partial response

Upregulation of PD-L1 by *EGFR* activation mediates the immune escape in *EGFR*-driven NSCLC, implicating an optional immune targeted therapy for NSCLC patients with *EGFR* mutation. In genetically engineered mouse models (GEMMs), *EGFR*-driven tumors express higher levels of PD-L1 with a more immunosuppressive tumor microenvironment (increased FoxP3+ T-cells, decreased CD8+/CD4+ ratio). The addition of an EGFR-TKI in these *EGFR*-mutant GEMM models modulated PD-L1 expression and reversed *EGFR*-pathway mediated immunosuppression. However, ICIs either alone or in combination with small molecule TKIs have low or inferior effect in *EGFR*-mutant or *ALK*-rearranged NSCLC. Therefore, GEMMs are not good models to study the effect of ICIs on human NSCLC tumors. As PD-L1 expression is lower in the majority of oncogene-driven NSCLC compared to non-oncogene-driven NSCLC, this may explain why the results in GEMMs did not correlate with the clinical observation. We also did not observe significant predictive or prognostic association between lymphocyte counts and correlation of PD-L1 or TMB. Using multiplex flow cytometry, we evaluated the immunophenotypic changes of PBMCs in a patient with *EGFR* E19 deletion who received 3 cycles of ICIs. We found that the ICI treatment increased lymphocytes, CD4+, CD4 terminally differentiated effector memory (TEMRA; CD45RA+ CCR7-), and CD8 naïve cells, and decreased all other types of immune subtype cells, including PD1 + CD4 and PD1 + CD8 cells. Of note, NSCLC patients with oncogene-driven mutations, such as *EGFR*, have been associated with hyperprogression to ICIs [66]. The impact of our observation and the association of blood circulating immune cells with TILs in TME in patients with oncogene-driven NSCLC after ICI treatment is unknown and deserves further exploration.

Malignant biofluids such as pleural effusion is a unique source for liquid biopsy that is currently underused for molecular diagnosis and tumor biology study. Pleural effusion affects at least 40% of patients with lung cancer. Up to 90% of these patients have confirmed malignant pleural effusion (MPE) and require palliative thoracentesis for symptomatic relief [67]. We explored the effect of TKIs on patient’s tumor cells from malignant pleural effusion and PBMCs using an in vitro co-culture model. We observed significant variations in individual’s response to different ICIs that were not clearly associated with tumor PD-L1 expression or sensitivity to targeted therapy with TKIs. To the best of our knowledge, this is the first study to determine the effect of small molecule targeted therapy on lymphocyte cells using NSCLC patient’s blood samples and malignant tumor cells. Ongoing study is determining the clinical utility of this in vitro assay in predicting clinical response to ICI therapy in patients with oncogene-driven NSCLC.

There are several limitations to this study, including its small sample size, it being a retrospective study, and no adjustment for multiplicity due to the exploratory nature. The potential selection bias and the imbalance of the baseline characteristics and treatment history in patients may have contributed to the treatment outcomes. Furthermore, dysregulation of various immunoregulatory cells and cytokines in the TME may be responsible for tumor response [68, 69]. We did not perform the functional analysis of immune subtypes in patient PBMCs collected before and after TKI or ICI treatment. Further study is needed to confirm our findings and understand the effect of TKI modulation on “cold” or “hot” tumor microenvironment.

## Conclusions

Molecularly targeted therapy by small molecule TKIs have various effects on modulating the blood immune cell count in patients with oncogene-driven NSCLC. There are unmet needs to understand the underpinning mechanisms and develop predictive biomarkers and assays to select the appropriate patients for ICI therapy.

## Data Availability

All data supporting the conclusions of this research article are included within the manuscript.

## Abbreviations

ALCs: Absolute lymphocyte counts
ALK: Anaplastic lymphoma kinase
ANC: Absolute neutrophil counts
ASH: American Society of Hematology
ASTCT: American Society for Transplantation and Cellular Theory
BTK: Bruton tyrosine kinase
CPB: Carboplatin, paclitaxel, bevacizumab
CR: Complete response
CTCAE: Common Terminology Criteria for Adverse Events
dNLR: derives neutrophil-to-lymphocyte ratio
EGFR: Epidermal growth factor receptor
GEP: Gene expression profile
EDTA: Ethylenediamine tetraacetic acid
GEMMs: Genetically engineered mouse models
HER2: Human epidermal growth factor receptor 2
ICIs: Immune checkpoint inhibitors
IHC: Immunohistochemistry
IRB: Institutional Review Board
LUAD: Lung adenocarcinoma
LUSC: Lung squamous cell carcinoma
mTOR: mammalian target of rapamycin
MPE: Malignant pleural effusion
MTS: 3-(4,5-dimethylthiazol-2-yl)-5-(3-carboxymethoxyphenyl)-2-(4-sulfophenyl)-2H-tetrazolium)
mNSCLC: metastatic non-small cell lung cancer
N: Number
NSCLC: Non-small cell lung cancer
NGS: Next generation sequencing
NCCN: National Comprehensive Cancer Network
NCI: National Cancer
NK: Natural killer
NOS: Not otherwise specified
OS: Overall survival
PBMCs: Peripheral blood mononuclear cells
PBS: Phosphate-buffered saline
PD: Progression disease
PD-L1: Programmed death-ligand 1
PFS: Progression free survival
PR: Partial response
RECIST: Response Evaluation Criteria in Solid Tumors
ROC: Receiver operating characteristic
SD: Stable disease
SD: Standard deviation
TCR: T-cell receptor
TKIs: Tyrosine kinase inhibitors
TILs: Tumor infiltrating lymphocytes
TMB: Tumor mutation burden
TME: Tumor micro environment
WBC: White blood cell
